# AmericasNLI: Machine translation and natural language inference systems for Indigenous languages of the Americas

**DOI:** 10.3389/frai.2022.995667

**Published:** 2022-12-02

**Authors:** Katharina Kann, Abteen Ebrahimi, Manuel Mager, Arturo Oncevay, John E. Ortega, Annette Rios, Angela Fan, Ximena Gutierrez-Vasques, Luis Chiruzzo, Gustavo A. Giménez-Lugo, Ricardo Ramos, Ivan Vladimir Meza Ruiz, Elisabeth Mager, Vishrav Chaudhary, Graham Neubig, Alexis Palmer, Rolando Coto-Solano, Ngoc Thang Vu

**Affiliations:** ^1^Department of Computer Science, University of Colorado Boulder, Boulder, CO, United States; ^2^Institute for Natural Language Processing, University of Stuttgart, Stuttgart, Germany; ^3^School of Informatics, University of Edinburgh, Edinburgh, United Kingdom; ^4^Courant Institute of Mathematical Sciences, New York University, New York, NY, United States; ^5^Institut für Computerlinguistik, University of Zurich, Zurich, Switzerland; ^6^Facebook AI Research, Menlo Park, CA, United States; ^7^URPP Language and Space, University of Zurich, Zurich, Switzerland; ^8^Institute of Computation, Universidad de la República, Montevideo, Uruguay; ^9^Department of Informatics, Universidade Tecnológica Federal do Paraná, Curitiba, Brazil; ^10^Universidad Tecnológica de Tlaxcala, Huamantla, Mexico; ^11^Department of Computer Science, Universidad Nacional Autónoma de México, Mexico City, Mexico; ^12^Facultad de Estudios Superiores Acatlán, Universidad Nacional Autónoma de México, Mexico City, Mexico; ^13^Microsoft Turing Research, Redmond, WA, United States; ^14^Language Technology Institute, Carnegie Mellon University, Pittsburgh, PA, United States; ^15^Department of Linguistics, University of Colorado Boulder, Boulder, CO, United States; ^16^Department of Linguistics, Dartmouth College, Hanover, NH, United States

**Keywords:** natural language processing, multilingual NLP, low-resource languages, natural language inference, machine translation, pretrained models, model adaptation

## Abstract

Little attention has been paid to the development of human language technology for truly low-resource languages—i.e., languages with limited amounts of digitally available text data, such as Indigenous languages. However, it has been shown that pretrained multilingual models are able to perform crosslingual transfer in a zero-shot setting even for low-resource languages which are unseen during pretraining. Yet, prior work evaluating performance on unseen languages has largely been limited to shallow token-level tasks. It remains unclear if zero-shot learning of deeper semantic tasks is possible for unseen languages. To explore this question, we present AmericasNLI, a natural language inference dataset covering 10 Indigenous languages of the Americas. We conduct experiments with pretrained models, exploring zero-shot learning in combination with model adaptation. Furthermore, as AmericasNLI is a multiway parallel dataset, we use it to benchmark the performance of different machine translation models for those languages. Finally, using a standard transformer model, we explore translation-based approaches for natural language inference. We find that the zero-shot performance of pretrained models without adaptation is poor for all languages in AmericasNLI, but model adaptation *via* continued pretraining results in improvements. All machine translation models are rather weak, but, surprisingly, translation-based approaches to natural language inference outperform all other models on that task.

## 1. Introduction

Languages with limited amounts of digitally available data, so-called low-resource languages, have recently started to receive increased attention from the natural language processing (NLP) community. Approaches targeted toward such languages include transfer learning techniques, such as pretraining (Devlin et al., 2019; Conneau et al., 2020) or zero-shot learning (Johnson et al., 2017), as well as machine translation-based solutions (Conneau et al., 2018; Fang et al., 2021; Ruder et al., 2021) or strategies based on alignment and projection (Yarowsky et al., 2001; Eskander et al., 2020). Which techniques are the most suitable typically depends on the use case and available resources. In order to, for instance, build a sentiment analysis system for Wixarika, an Indigenous language spoken in Mexico with no annotated data for a sentiment analysis task, one could pretrain a model on unlabeled text in English and Wixarika and then finetune it using English sentiment analysis data (a zero-shot approach) or, assuming one has a suitable machine translation (MT) system, translate the Wixarika data into English and, in a straightforward fashion, apply an English sentiment analysis system.

However, for many state-of-the-art techniques, it is not obvious if they are actually applicable to extremely low-resource languages, which are sometimes also called *truly* low-resource languages in the literature (Agić et al., 2016). In particular, many techniques targeted at circumventing a lack of data for a given task–language combination still require resources that are frequently not available for the large majority of the world's roughly 7,000 languages, namely sufficient amounts of unlabeled (digitally available) data or MT systems with a sufficiently good performance. Prior work explored which model adaptation techniques—i.e., algorithms that adapt large pretrained models to a language which has not been part of its pretraining data—result in the best zero-shot performance when only limited amounts of raw text are available (Ebrahimi and Kann, 2021). Surprisingly, they found that the most straightforward approach, a continuation of the original masked language pretraining on target-language data, performs best, even when compared to more complicated techniques. Yet, this prior analysis was limited by the fact that the only available multilingual test data for truly low-resource languages consisted exclusively of token-level tasks: named entity recognition (NER) and part-of-speech (POS) tagging.

Here, we describe the creation of a multilingual dataset aimed at enabling the evaluation of existing techniques for a higher-level semantic task in truly low-resource languages: AmericasNLI, a multiway parallel natural language inference (NLI) dataset in 10 Indigenous languages of the Americas: Asháninka, Aymara, Bribri, Guarani, Nahuatl, Otomí, Quechua, Rarámuri, Shipibo-Konibo, and Wixarika. The amount of digitally available raw text in the languages in AmericasNLI is limited—as is data to train MT systems. Furthermore, as we describe in Section 2.1.3, the typological properties of those languages are quite different from many high-resource languages, such as, importantly for this work, English and Spanish. AmericasNLI is multiway parallel: in addition to enabling the evaluation of NLI models, it thus also makes benchmarking of MT systems possible. Leveraging this newly created dataset, we give initial answers to the following research questions (RQs): (1) Do zero-shot approaches for NLI, a high-level reasoning task, based on model adaptation perform well for truly low-resource languages? (2) How do translation-based approaches work for NLI if all we have are poor MT systems, and how do we build initial MT systems for the AmericasNLI languages? (3) Finally, how do the two different strategies (zero-shot vs. translation-based) compare?

Regarding RQ1, we explore the performance of XLM-R (Conneau et al., 2020) with and without continued pretraining. For RQ2, we survey multiple systems resulting from the recent AmericasNLP 2021 Shared Task on Open Machine Translation (Mager et al., 2021), before employing a transformer (Vaswani et al., 2017) model within a translation-based approach to NLI. Finally, considering RQ3, we compare the two different strategies.

Overall, we find that XLM-R's non-adapted zero-shot performance is close to random guessing, which differs from comparable results for NER and POS tagging (Ebrahimi and Kann, 2021). However, in line with previous findings for token-level tasks, model adaptation *via* continued pretraining does result in consistent improvements. However, surprisingly, the best results are obtained by translation-based approaches, even with MT systems as weak as those available for the Indigenous languages in AmericasNLI.

**Related Work on Pretrained Multilingual Models:** Before the advent of transformers (Vaswani et al., 2017) and, subsequently, pretrained transformer models, crosslingual transfer was often achieved through word embeddings (Mikolov et al., 2013; Pennington et al., 2014; Bojanowski et al., 2017), either by aligning monolingual embeddings into the same embedding space (Grave et al., 2018; Lample et al., 2018a,b) or by training multilingual embeddings (Ammar et al., 2016; Artetxe and Schwenk, 2019). Pretrained multilingual models can be considered a contextualized version (Peters et al., 2018) of multilingual embeddings: the vector representation of each word (or subword) depends on the sentence context. Unlike traditional word embeddings, these models follow the standard pretraining–finetuning paradigm: they are first trained on unlabeled monolingual corpora from various languages and later finetuned on target-task data in a—usually high-resource—source language, before being applied to target-language data. The first large multilingual model was mBERT (Devlin et al., 2019), which is pretrained on Wikipedia data from 104 languages using masked language modeling (MLM) and next sentence prediction (NSP) as its training objectives. XLM-R (Conneau et al., 2020) is an improved version of mBERT, which is trained on data from 100 different languages using only the MLM objective.

Out-of-the-box pretrained models show poor zero-shot performance on languages that are not part of their pretraining data (and not similar to any of the pretraining languages). Multiple methods have been proposed to adapt models to unseen languages, including extending the vocabulary, transliterating the target text, and continuing pretraining before finetuning (Chau et al., 2020; Lauscher et al., 2020; Muller et al., 2020; Pfeiffer et al., 2020a,b; Wang et al., 2020). However, with very few exceptions (e.g., Ebrahimi and Kann, 2021), prior work assumes large amounts of data for adaptation; those are not available for many truly low-resource languages, such as the Indigenous languages we consider in this work. In addition, adaptation methods are generally evaluated on shallow token-level tasks such as POS tagging or NER. AmericasNLI makes it possible to evaluate such adaptation methods on tasks that require a better understanding of sentence semantics.

**Related Work on Natural Language Inference:** The largest and most widely used datasets for NLI in English are SNLI (Bowman et al., 2015) and MNLI (Williams et al., 2018). XNLI Conneau et al. (2018) is the multilingual expansion of MNLI to 15 languages: it consists of manually translated evaluation sets and machine-translated training sets. While datasets for NLI or the similar task of recognizing textual entailment exist for other languages (Bos et al., 2009; Alabbas, 2013; Eichler et al., 2014; Amirkhani et al., 2020), their lack of similarity prevents a generalized study of crosslingual zero-shot performance. In contrast, all examples in XNLI are 15-way parallel. To extend this property of XNLI to Indigenous languages we choose to translate the Spanish subcorpus of XNLI when building AmericasNLI as opposed to creating examples directly in the target language.

## 2. Materials and methods

### 2.1. The Americasnli dataset

#### 2.1.1. Motivation

Recently, prior work by Ebrahimi and Kann (2021) explored the question of how a pretrained language model—namely XLM-R (Conneau et al., 2020)—can be adapted to so-called unseen languages, i.e., languages that do not form part of the model's pretraining data. A comparison of different techniques showed that continued pretraining (Chau et al., 2020) with either a masked language modeling objective or a translation language modeling objective results in the best models for a large set of languages.

However, Ebrahimi and Kann (2021) was limited to two tasks, part-of-speech (POS) tagging and named entity recognition (NER). The former consists of assigning syntactic categories—such as *noun, verb*, or *adjective*—to words within a sentence context. The latter consists of identifying named entities—e.g., *persons* or *locations*—within a sentence and, in its most common version, assigning a tag to each word which indicates if this word is part of a named entity or not. Those two tasks are not representative of the diverse variety of NLP tasks we eventually want to develop systems for. Importantly, POS tagging and NER are different from many other NLP tasks in the following ways:

- They are *token-level* or *sequence tagging* tasks, as shown in Table 1.
- They can be solved at a high accuracy from *character-level clues alone*: e.g., the suffix *-ing* is a strong indication of a verb in English.
- They require, importantly, *limited to no understanding of semantics or reasoning abilities*.

**Table 1:** An English example sentence with corresponding POS tags (line 2) and NER tags (line 3).

	Jen	Must	Draw	Attention	To	The	Distribution	Of	This	Form	In	Those	Dialects	.
POS Tags	NP	AUX	VERB	NOUN	ADP	DET	NOUN	ADP	DET	NOUN	ADP	DET	NOUN	PCT
NER Tags	B-PERS	O	O	O	O	O	O	O	O	O	O	O	O	O

Thus, prior work leaves the question open if—and which—state-of-the-art approaches are applicable to higher-level semantic tasks, which cannot be solved by looking at individual words and which require at least some understanding of the meaning of longer chunks of text. According to Bowman et al. (2015), “Understanding entailment and contradiction is fundamental to understanding natural language, and inference about entailment and contradiction is a valuable testing ground for the development of semantic representations.” Thus, the task of NLI is a suitable test bed for the higher-level semantic abilities of NLP models. In its typical formulation, NLI consists of, given a sentence pair—the *premise* and the *hypothesis*—, predicting the relationship between the two sentences as either *entailment, neutral*, or *contradiction*. An English example for the *entailment* label is shown in line 2 of Table 2.

**Table 2:** A parallel example in AmericasNLI; label: *entailment*.

**Language**	**Premise**	**Hypothesis**
en	And he said, Mama, I'm home.	He told his mom he had gotten home.
es	Y él dijo: Mamá, estoy en casa.	Le dijo a su madre que había llegado a casa.
aym	Jupax sanwa: Mamita, utankastwa.	Utar purinxtwa sasaw mamaparux sanxa
bzd	Ena ie' iche: ãmì, ye' tso' ù a.	I ãmì a iché irir tö ye' démine ù a.
cni	Iriori ikantiro: Ina, nosaiki pankotsiki.	Ikantiro iriniro yaretaja pankotsiki.
gn	Ha ha'e he'i: Mama, aime ógape.	He'íkuri isýpe o9Duahêhague hógape.
hch	metá mik+ petay+: ne mama kitá nepa yéka.	yu mama m+pa+ p+ra h+awe kai kename yu kitá he nuakai.
nah	huan yehhua quiihtoh: Nonantzin, niyetoc nochan	quiilih inantzin niehcoquia
oto	xi nydi biênâ: maMe dimi an ngû	bimâbi o ini maMe guê o ngû
quy	Hinaptinmi pay nirqa: Mamay wasipim kachkani.	Wasinman chayasqanmanta mamanta willarqa.
shp	Jara neskata iki: tita, xobonkoriki ea.	Jawen tita yoiaia iki moa xobon nokota.
tar	Alí je aníli échiko: ku bitichí ne atíki Nana	Iyéla ku ruyéli, mapu bitichí ku nawáli.

However, no NLI dataset exists for languages which are not part of XLM-R's pretraining data. Thus, in order to make such an evaluation possible, we build AmericasNLI, an NLI dataset in truly low-resource languages, which are *unseen* to state-of-the-art pretrained models: Asháninka, Aymara, Bribri, Guarani, Nahuatl, Otomí, Quechua, Rarámuri, Shipibo-Konibo, and Wixarika (cf. Section 2.1.3). Additionally, we build the dataset in such a way that all sentences are multiway parallel—this enables us to leverage it for a second purpose: evaluating the performance of MT systems for these 10 truly low-resource languages.

In the following, we will describe the data collection process (Section 2.1.2) and provide more details on the languages (Section 2.1.3).

#### 2.1.2. Data collection

In order to make it possible to evaluate systems for both NLI and MT with our data, we translate an existing dataset into Indigenous languages as opposed to creating a new dataset from scratch. The dataset we translate is a subset of XNLI (Conneau et al., 2018), which, in turn, is a multilingual version of the MNLI (Williams et al., 2018) development and test sets.

It is easier to find translators between the languages we are interested in and Spanish than English. Hence, we translate from the Spanish version of XNLI. Furthermore, code-switching is often used to describe certain topics, and, while many words without an exact equivalence in the target language are worked in through translation or interpretation, others are kept in Spanish. To minimize the amount of Spanish vocabulary in the translated examples, we choose sentences from genres which we expect to be relatively easy to translate into the target languages. Our final dataset consists of translations of the “face-to-face,” “letters,” and “telephone” genres in XNLI. We choose up to 750 examples from each of the development and test set; the exact counts for each language appear in Table 3.

**Table 3:** Distribution of labels in the test and development sets, per language.

**Language**	**Split**	**Entailment**	**Contradiction**	**Neutral**	**Majority Baseline**
aym	Test	250	250	250	0.333
	Dev	248	248	247	0.334
bzd	Test	250	250	250	0.333
	Dev	248	248	247	0.334
cni	Test	250	250	250	0.333
	Dev	220	220	218	0.334
gn	Test	250	250	250	0.333
	Dev	248	248	247	0.334
hch	Test	250	250	250	0.333
	Dev	248	248	247	0.334
nah	Test	246	245	247	0.335
	Dev	193	195	197	0.337
oto	Test	249	249	250	0.334
	Dev	78	75	69	0.351
quy	Test	250	250	250	0.333
	Dev	248	248	247	0.334
shp	Test	250	250	250	0.333
	Dev	248	248	247	0.334
tar	Test	250	250	250	0.333
	Dev	248	248	247	0.334

Following the process used during the creation of XNLI, we translate sentences individually, i.e., translators do not have access to the entire pair that makes up an NLI example. While we expect this to make the translations slightly more natural, this also has the potential to, for a small number of examples, invalidate the original label. We acknowledge that this is a source of noise in our experiments, but, due to difficulties finding native speakers of our 10 languages who are available to work on this task, we leave a manual verification of the AmericasNLI labels to future work.

#### 2.1.3. Languages

**Aymara (aym)** is an Indigenous language, which is spoken in Bolivia, Chile, and Peru by more than two million people (Homola, 2012). Multiple dialectal variants exist, including Northern Aymara, which is spoken on the southern Peruvian shore of Lake Titicaca as well as around La Paz and Southern Aymara, which is spoken in the eastern half of the Iquique province in northern Chile, the Bolivian department of Oruro, in northern Potosi, and southwest Cochabamba. However, Southern Aymara is slowly being replaced by Quechua in the last two regions. AmericasNLI examples are translated into the Central Aymara variant, specifically Aymara La Paz. Aymara is a polysynthetic language and follows an SOV word order. A rare linguistic phenomenon found in this language is vowel elision.

**Asháninka (cni)** is an Indigenous language from the Amazonian region, which belongs to the Arawak family and has around 74,000 speakers^1^ in Central and Eastern Peru, in a geographical region located between the eastern foothills of the Andes and the western fringe of the Amazon basin (Mihas, 2017). While Asháninka in a strict sense refers to the linguistic varieties spoken in Ene, Tambo and Bajo Perené rivers, the name is also used to talk about the following nearby and closely-related Asháninka varieties: Alto Perené, Pichis, Pajonal, Ucayali-Yurua, and Apurucayali. Although it is the most widely spoken Amazonian language in Peru, certain varieties, such as Alto Perené, are highly endangered. Asháninka is agglutinative and polysynthetic and follows a VSO word order. The verb is the most morphologically complex word class, with a rich repertoire of aspectual and modal categories. The language lacks case, except for one locative suffix, and the grammatical relations of subject and object are indexed as affixes on the verb itself. Other notable linguistic features of the language include obligatory marking of a realis/irrealis distinction on the verb, a rich system of applicative suffixes, serial verb constructions, and a pragmatically conditioned split intransitivity. Code-switching with Spanish or Portuguese is a regular practice in everyday dialogue.

**Bribri (bzd)** is a Chibchan language, which is spoken by only around 7,000 people in Southern Costa Rica^2^ (INEC, 2011). There are three known dialectal variants. Bribri has only been a written language for about 40 years, which is why existing materials have a large degree of idiosyncratic variation. These variations are standardized in AmericasNLI, which is written in the Amubri variant.While Bribri is still spoken by children, it is currently a vulnerable language (Moseley, 2010; Sánchez Avendaño, 2013). It does not have official status and it is not the main medium of instruction of Bribri children, but it is offered as a class in primary and secondary schools. Bribri is a tonal and fusional language with an SOV word order. Its grammar also includes phenomena like head-internal relative clauses, directional verbs and numerical classifiers (Murillo and Victoria, 2018a). There are several orthographies which use different diacritics for the same phenomena. Furthermore, the dialects of Bribri differ in their exact vocabularies, and there are phonological processes, like the deletion of unstressed vowels, which also change the tokens found in texts.

**Guarani (gn)** is an Indigenous language spoken by 6–10 million people in South America. Around 3 million people use Guarani as their main language, including members of more than 10 native nations in Paraguay, Brazil, Argentina, and Bolivia, along with Paraguayan, Argentinian, and Brazilian peoples. According to the Paraguayan Census, there were around 1.35 million monolingual speakers in 2002, and this number has since increased to around 1.5 million people (Melià, 1992; Dos Santos, 2017)^3^. Although the use of Guarani as a spoken language is much older, the first written record dates to 1591 (Catechism) followed by the first dictionary in 1639 and linguistic descriptions in 1640. Guarani usage in text continued until the Paraguay-Triple Alliance War (1864–1870) and declined thereafter. However, from the 1920s on, Guarani has slowly reemerged and received renewed focus. In 1992, Guarani was the first American language declared an official language of a country, followed by a surge of recognition in the early 21st century^4^. The official grammar of Guarani was approved in 2018. Guarani is an agglutinative language with an SVO word order. Code-switching with Spanish or Portuguese is common among speakers.

**Nahuatl (nah)** Nahuatl is an Indigenous language belonging to the Nahuan subdivision of the Uto-Aztecan language family. There are 30 recognized variants of Nahuatl spoken by over 1.5 million speakers across 17 different states of Mexico, where Nahuatl is recognized as an official language (SEGOB, 2020b). Nahuatl is polysynthetic and agglutinative. Most sentences follow an SVO word order, but a VSO order can be used for contrast and focus, and an SOV order can express emphasis (MacSwan, 1998). The examples in AmericasNLI belong to the Central Nahuatl (Náhuatl de la Huasteca) dialect. As there is a lack of consensus regarding the orthographic standard, for AmericasNLI the orthography has been normalized to a version similar to Classical Nahuatl.

**Otom**í **(oto)** is part of the Oto-Pamean language family. Nine linguistic variants exist, which have different regional self-denominations, such as ñ*ähñu* or ñ*ähño, hñähñu, ñuju, ñoju, yühu, hnähño, ñühú, ñanhú, ñöthó, ñható* and *hñothó*^5^. Otomí has around 308,000 speakers, who are living across 7 Mexican states. In the state of Tlaxcala, the *yuhmu* or ñ*uhmu* variant is spoken by fewer than 100 speakers, and we use this variant for the Otomí examples in AmericasNLI. Otomí is a tonal language following an SVO word order, and many words are homophonous to Spanish (Cajero, 1998, 2009).

**Quechua (quy)**, which is also called *Runasimi*, is an Indigenous language family spoken by the Quechua peoples who live primarily in the Peruvian Andes. With around 8–10 million speakers it is one of the most widely spoken pre-Columbian language families of the Americas, and approximately 25% (7.7 million) of Peruvians speak a Quechuan language. Historically, Quechua was the main language family during the Incan Empire, and it was spoken until the Peruvian struggle for independence from Spain in the 1780s. Currently, many variants of Quechua are widely spoken and it is the co-official language of many regions in Peru. The subdivisions of Quechua include Southern, Northern, and Central Quechua. The examples in AmericasNLI are translated into the standard version of Southern Quechua, which is known as Quechua Chanka or Quechua Ayacucho. This variant is spoken in different regions of Peru, but can also be understood by people in different areas of other countries, such as Bolivia or Argentina.

**Rarámuri (tar)** is an Indigenous language that is also known under the name *Tarahumara*, which means *light foot*^6^. Rarámuri is part of the Taracahitan subgroup of the Uto-Aztecan language family (Goddard, 1996). It is an official language of Mexico, spoken mainly in the Sierra Madre Occidental region in the state of Chihuahua by a total of around 90,000 speakers (SEGOB, 2020c). There are five variants of Rarámuri. AmericasNLI examples are translated into the Highlands variant^7^, and translation orthography and word boundaries are similar to Caballero (2008). Rarámuri is an agglutinative and polysynthetic language, which is characterized by a head-marking structure (Nichols, 1986), and follows an SOV word order.

**Shipibo-Konibo (shp)** is a Panoan language spoken by around 35,000 native speakers in the Amazonian region of Peru. It is a language with agglutinative processes, the majority of which are suffixes. However, clitics are also used and are a widespread element in Panoan literature (Valenzuela, 2003). Shipibo-Konibo follows an SOV word order (Faust, 1973) and uses postpositions (Vasquez et al., 2018). The translations in AmericasNLI employ the official alphabet and follow the standard writing supported by the Ministry of Education in Peru.

**Wixarika (hch)** is an Indigenous language and is also called *Huichol* by its speakers, which translates to *the language of the doctors and healers* (Lumholtz, 2011). Is is part of the Corachol subgroup of the Uto-Aztecan language family (Campbell, 2000). Wixarika is a national language of Mexico with four variants: Northern, Southern, Eastern, and Western^8^. It is spoken mainly in the three Mexican states of Jalisco, Nayari, and Durango by a total of around 48,000 speakers (SEGOB, 2020a). Translations in AmericasNLI are in Northern Wixarika and use an orthography common among native speakers (Mager-Hois, 2017). Wixarika is a polysynthetic language with head-marking (Nichols, 1986), a head-final structure (Greenberg, 1963), nominal incorporation, argumentative marks, inflected adpositions, possession marks, as well as instrumental and directional affixes (Iturrioz and Gómez-López, 2008). It follows an SOV word order, and lexical borrowing from as well as code-switching with Spanish are common.

### 2.2. Machine translation for Indigenous languages of the Americas

One of the use cases of AmericasNLI is to benchmark the performance of MT systems between Indigenous languages of the Americas and a high-resource language^9^. To this aim, AmericasNLI was featured in the AmericasNLP 2021 Shared Task on Open Machine Translation (Mager et al., 2021). We now survey different modeling and training techniques for MT between the AmericasNLI languages and Spanish that have been explored in the context of this competition. The exact hyperparameters for all systems we are discussing here can be found in the respective system description papers (references are listed in **Table 5**). Then, as AmericasNLI only encompasses a development and test set, we describe publicly available out-of-domain training sets for the languages in AmericasNLI. We focus on translation *from* Spanish *into* an Indigenous language and leave the opposite direction to future work.

#### 2.2.1. Models

**[M1] Word-based Statistical Machine Translation (SMT):** As the sizes of available training sets for MT between Spanish and the AmericasNLI languages are limited, an SMT model might be a suitable choice. Here, we report the performance of an IBM Model 2 (Brown et al., 1993).

**[M2] Transformer Sequence-to-Sequence Model:** Another approach, which is common among AmericasNLP 2021 Shared Task submissions, is a state-of-the-art neural MT approach, a transformer sequence-to-sequence (seq2seq) model (Vaswani et al., 2017). This model consists of a transformer encoder, which takes an input sentence and represents it as a sequence of continuous vectors, in combination with a transformer decoder, which is a language model conditioned on the encoder output.

**[M3] Ensembles:** A common strategy to improve system performance is to create an ensemble (Bojar et al., 2014) of multiple individual models, i.e., to combine them into a single prediction. Here, in order to ensemble, output probabilities at each time step are averaged across models.

#### 2.2.2. Training techniques

**[TT1] Pretraining: Denoising (T5-style):** Pretraining a seq2seq model with a denoising objective, as proposed for T5 (Raffel et al., 2020), consists of, given a sentence with one or more masked inputs, generating the original sentence. 15% of the original tokens are selected randomly and masked out, and consecutive selected tokens are substituted by a single mask.

**[TT2] Pretraining: Denoising (mBART-style):** We further report results of denoising instances with both masking and permuted sentences as proposed by Liu et al. (2020). Thirty-five percent of the original tokens are selected randomly and masked out, and, again, consecutive selected tokens are substituted by a single mask. As the dataset consists of multiple languages, special language ID tokens are used.

**[TT3] Backtranslation:** Backtranslation (Sennrich et al., 2016) consists of using a preliminary MT model to translate monolingual text in the target language, i.e., the Indigenous language in our case, into the source language to create pseudo-parallel data. This data is then used—typically in combination with the original training data—to further train the preliminary MT system or to train a new MT system from scratch. To improve performance, the pseudo-parallel data is filtered to remove low-quality examples; for details we refer the reader to Vázquez et al. (2021).

**[TT4] Multilingual Translation Training:** We further compare training on multiple language pairs simultaneously in a multitask fashion, either during pretraining or finetuning. We explore different combinations of AmericasNLI languages, Spanish, English, and other languages. During multilingual training, the target language is indicated with a special token, following Johnson et al. (2017).

**[TT5] Single-Language Pair Translation Finetuning:** The standard approach to obtain final MT systems for individual language pairs is to (optionally after pretraining) finetune on a single language pair per model.

#### 2.2.3. Parallel training data

All systems are trained on the parallel training data provided for the AmericasNLP 2021 Shared Task on Open Machine Translation. It consists of parallel text between Spanish and the Indigenous languages from multiple sources; all sources are out-of-domain with respect to the AmericasNLI development and test sets. We describe the data for all languages in the following; statistics are shown in Table 4.

**Table 4:** Overview of the sizes of the training sets provided for the AmericasNLP 2021 Shared Task on Open Machine Translation (Mager et al., 2021).

**Asháninka**	**Aymara**	**Bribri**	**Guarani**	**Nahuatl**	**Otomí**	**Quechua**	**Rarámuri**	**Shipibo-Konibo**	**Wixarika**
3,883	6,531	7,508	26,032	16,145	4,889	125,008	14,721	14,592	8,966

The datasets are coming from different distributions than the AmericasNLI development and test set, making AmericasNLI an out-of-domain MT task.

**Spanish–Aymara:** The Spanish–Aymara training set is obtained from Global Voices (Prokopidis et al., 2016) and published in OPUS (Tiedemann, 2012). This text is from a similar variant of Aymara as AmericasNLI, but shows differences in writing styles.

**Spanish–Asháninka:** The parallel training data for Spanish–Asháninka is obtained by collecting texts from different domains such as traditional stories, educational texts, and environmental laws for the Amazonian region (Romano et al., 2008; Mihas, 2011; Ortega et al., 2020). One peculiarity of Asháninka is that there are many neologisms that are not spread to the speakers of all communities. During the translation of the development and test sets, only words and concepts that are generally well known are translated, while other terms are preserved in Spanish.

**Spanish–Bribri:** The training set for Spanish–Bribri is obtained from six sources (Constenla et al., 2004; Enrique, 2005; Jara Murillo and García Segura, 2013; Flores Solórzano, 2017; Murillo and Victoria, 2018a,b; Feldman and Coto-Solano, 2020), including a dictionary, a grammar, two language learning textbooks, one storybook and the transcribed sentences from one spoken corpus. Those texts are written in Amubri, Coroma and Salitre, three of the main Bribri dialects. All training sentences come from similar domains: either from traditional stories or language learning examples. The development and test sentences are translated by a speaker of the Amubri dialect.

**Spanish–Guarani:** The training corpus for Spanish–Guarani (Chiruzzo et al., 2020) is collected from web sources (blogs and news articles) that contain a mix of dialects, from pure Guarani to a Guarani that makes strong use of Spanish neologisms. The development and test corpora, on the other hand, are in standard Paraguayan Guarani.

**Spanish–Nahuatl:** The Spanish–Nahuatl training corpus comes from Gutierrez-Vasques et al. (2016) and has considerable dialectal, domain, orthographic and diachronic variation. However, the majority of sentences are closer to a Classical Nahuatl orthographic “standard.” The development and test sets were translated to modern Nahuatl, and, in order to be more similar to the training corpus, an orthographic normalization was applied. A simple rule based approach was used, which was based on the most predictable orthographic changes between modern varieties and Classical Nahuatl.

**Spanish–Otom**í**:** The training set for Spanish–Otomí^10^ comes from a set of different sources and, thus, represents more than one dialectal variant and orthographic standard. However, most of the texts belong to the Valle del Mezquital dialect, while the development and test sets are from the Ñûhmû de Ixtenco, Tlaxcala, variant, which also has its own orthographic system.

**Spanish–Quechua:** The texts in the training set for Spanish–Quechua are written in the Quechua Cuzco and Quechua Ayacucho variants and are taken from JW300 (Agić and Vulić, 2019). JW300 consists of Jehovah's Witness texts, sentences extracted from the official dictionary of the Minister of Education (MINEDU), and miscellaneous dictionary entries and samples which have been collected and reviewed by Huarcaya Taquiri (2020). The development and test sets are translated into Quechua Ayacucho.

**Spanish–Rarámuri:** The parallel Spanish–Rarámuri training data consists of a set of extracted phrases from the Rarámuri dictionary (Brambila, 1976). However, we could not find any description of the dialectal variant to which these examples belong. The development and test set are translations from Spanish into the highlands Rarámuri variant and may differ from the training set.

**Spanish–Shipibo-Konibo:** The training sets for Spanish–Shipibo-Konibo are taken from different sources and translators, including translations of a sample from the Tatoeba dataset (Gómez Montoya et al., 2019), translated sentences from books for bilingual education (Galarreta et al., 2017), and dictionary entries and examples (Loriot et al., 1993). The development and test sets are created following the official convention, as are the training sets.

**Spanish–Wixarika:** The training data for Spanish–Wixarika is taken from Mager et al. (2018a) and is a translation of the fairy tales of Hans Christian Andersen. The training, development and test sets are all written in the same dialectal variation, Wixarika of Zoquipan, and use the same orthography. However, word boundaries are not always marked according to the same criteria in the training set and AmericasNLI.

**Additional Data:** Some systems are trained on additional parallel data that has been collected by the shared task participants. These datasets include translations in the JHU Bible Corpus (McCarthy et al., 2020), JW300 (Agić and Vulić, 2019), and resources available in HTML or PDF format, such as constitutions, poems, lyrics, and educational materials.

#### 2.2.4. Monolingual training data

Monolingual data in the AmericasNLI languages from a variety of sources are used by AmericasNLP 2021 Shared Task (Mager et al., 2021) participants to improve MT performance *via* pretraining and backtranslation. Specifically, we report results from systems using the following monolingual resources: Wikipedia (for Aymara, Guarani, Nahuatl, and Quechua), text from the OPUS corpus collection (for Aymara, Guarani, Hñähñu, Nahuatl and Quechua), monolingual Bible editions, and fiction and non-fiction books in the languages.

### 2.3. Natural language understanding for Indigenous languages of the Americas

#### 2.3.1. Pretraining

As with other areas of NLP, the pretraining–finetuning framework, along with the use of large transformer models has become the de facto standard approach for crosslingual transfer. This overtakes prior approaches, which relied mainly on either the alignment of monolingual embeddings or the creation of non-contextual multilingual representations. Multilingual transformers are a natural extension of their monolingual counterpart: rather than simply training on an unlabeled monolingual corpus, data from multiple languages is used. This extension also applies to the creation of the vocabulary, which consists of subword units created while considering all languages simultaneously. The most commonly used multilingual models are multilingual BERT (mBERT) (Devlin et al., 2019) and XLM-Roberta (XLM-R) (Conneau et al., 2020). mBERT is an identical model, in architecture, to the original BERT model, and has been pretrained using a masked language modeling (MLM) and next sentence prediction (NSP) objective. mBERT utilizes data from the 104 largest Wikipedia corpora. Analogously, XLM-R is a multilingual model based on the RoBERTa architecture. It uses SentencePiece (Kudo and Richardson, 2018) tokenization, with a vocabulary of 250k subwords. While XLM-R is only trained with an MLM objective, its predecessor, XLM, was additionally trained with a translation language modeling (TLM) (Conneau and Lample, 2019) objective, which relies on the usage of sentence aligned data. Along with minor modifications, the TLM objective extends the MLM objective by allowing the model to predict masked tokens using not only the context within the sentence, but also the tokens found in the parallel sentence. In our work, we focus on **XLM-R** as the underlying language model for our experiments.

One benefit of these pretrained models is the ease of use, particularly for zero-shot transfer. For this approach, it is assumed that, for a given supervised task, no labeled data is available in the language of interest. However, we can leverage knowledge from labeled data in a different language; after finetuning the model on this data, we can directly apply it to the target language, without any explicit alignment or projection.

#### 2.3.2. Model adaptation

While multilingual pretrained models provide an elegant approach to zero-shot transfer, the best downstream performance is generally reserved for the languages with the largest representation within the original pretraining corpus. As pretraining time and vocabulary size is finite, more parameter updates and subwords will be dedicated to these languages; as such, the expected performance for a language decreases as its representation (or the representation of a language sufficiently similar to it^11^) decreases. As the languages most likely to have low representation in the model are generally low-resource, a situation arises where performance is lacking precisely for the languages which require it most.

Fortunately, the performance for a target language can be improved through model adaptation, which leverages unlabeled data in an intermediate step between the original pretraining and final finetuning. This is analogous to domain adaptation, which aims to prepare a model for finetuning in a previously unseen domain (e.g., finetuning a general English model on medical data). As unlabeled data is much easier to acquire, this approach becomes viable even for low-resource languages. Various adaptation methods have been proposed, each focusing on different parts of the model. Continued pretraining is the simplest approach, which just continues training with the original unsupervised learning objective on data in the target language (or on parallel data when available). Other approaches include vocabulary adaptation (Wang et al., 2020), which can modify or add the subword vocabulary in order to handle unseen scripts and improve the fragmentation ratio for a specific language, which has been shown to correlate with better performance. Adapters (Pfeiffer et al., 2020b) are additional layers which can be introduced to the model and trained independently, while the original parameters are frozen. Training adapters on both task-specific and language-specific data allow for a lightweight approach toward adaptation, which can rival full finetuning in some cases.

Prior work has shown that **continued pretraining** is likely the most reliable adaptation approach for low-resource languages, as it does not introduce any new, randomly initialized parameters into the model (Ebrahimi and Kann, 2021). As such, we focus on this approach for our experiments.

#### 2.3.3. Translation-based approaches

Outside of a pure zero-shot approach to crosslingual transfer, one can also leverage parallel data to use a translation based approach. Here, the parallel data is used to train a translation model, and for finetuning one either translates the training examples to match the evaluation language (translate-train), or conversely, translates the test examples to match the training examples (translate-test).

## 3. Results

### 3.1. Machine translation results

The results for most systems submitted to the AmericasNLP Shared Task on Open Machine Translation, together with the most relevant system properties, are shown in Table 5: we omit the “random babbling” baseline by Bollmann et al. (2021) as well as all systems trained on parts or all of the development set from this analysis. Due to the nature of the shared task we summarize here, systems are trained on different datasets and not directly comparable; we focus on general trends and leave a principled comparison of the effects and interactions of model architectures, training techniques, and datasets to future work.

**Table 5:** MT results for systems using different model architectures and training techniques, as submitted to the AmericasNLP 2021 Shared Task on Open Machine Translation (Mager et al., 2021).

**Reference**	**System**	**M1**	**M2**	**M3**	**TT1**	**TT2**	**TT3**	**TT4**	**TT5**	**Data**	**aym**	**bzd**	**cni**	**gn**	**hch**	**nah**	**oto**	**quy**	**shp**	**tar**
Mager et al. (2021)	1		✓						✓		15.7	6.8	10.2	19.3	12.6	15.7	5.4	30.4	12.1	3.9
Vázquez et al. (2021)	4		✓					✓		+	21.6	13.0	23.6	27.6	25.4	24.3	14.1	25.2	29.4	15.5
Vázquez et al. (2021)	5		✓				✓	✓		+	**28.3**	**16.5**	**25.8**	**33.6**	**30.4**	**26.6**	**14.7**	34.3	**32.9**	**18.4**
Knowles et al. (2021)	1		✓	✓^*hch*^				✓	✓		–	–	–	26.1	26.4	23.7	–	–	–	14.3
Moreno (2021)	1		✓					✓	✓	+	–	–	–	–	–	–	–	**34.6**	–	–
Parida et al. (2021)	1	✓							✓		20.2	11.3	25.3	17.2	21.4	21.8	11.8	24.4	20.4	15.5
Parida et al. (2021)	2		✓						✓		16.6	3.7	13.0	10.8	9.4	11.2	1.4	23.2	8.9	2.8
Parida et al. (2021)	3		✓					✓	✓		19.4	13.2	18.6	18.7	20.6	17.4	11.0	26.3	14.9	8.4
Parida et al. (2021)	4		✓						✓		–	–	–	–	–	14.5	–	–	–	8.9
Parida et al. (2021)	5		✓						✓		15.1	10.6	17.4	20.7	16.9	16.6	7.4	27.3	12.5	7.3
Nagoudi et al. (2021)	1		✓		✓				✓	+	17.8	11.2	17.8	–	19.4	19.5	8.2	–	12.4	10.2
Nagoudi et al. (2021)	3		✓		✓				✓	+	18.2	11.0	17.6	–	18.6	18.8	8.1	–	–	10.2
Zheng et al. (2021)	3		✓			✓			✓	+	20.9	13.1	21.4	25.4	22.9	23.6	12.5	32.8	17.5	12.3

Translations are from Spanish into the indicated Indigenous language. Best results per language pair are shown in bold. Model architectures (M1–M3) and training teachniques (TT1–TT5) are described in Subsection 2.2. ‘+' in the *Data* column indicates that additional data besides the AmericasNLP 2021 Shared Task training set is used.

**Absolute Performances:** As this is an out-of-domain translation task—i.e., the training set on the one hand and development and test sets on the other come from different distributions—it is very challenging. The best systems only obtain a ChrF score between 34.6 (for quy) and 14.7 (for oto). Results reported by Mager et al. (2021) further show that including the development set during training has a huge effect on performance. This is a strong indication that the out-of-domain setting contributes to this MT task's difficulty.

**Model Architectures:** Transformer seq2seq models (M2) are used for most systems with small variations in their hyperparameter settings. While this indicates that transformer models are reasonable choices for MT of the AmericasNLI languages, this leaves open the question of how other neural models, such as LSTM seq2seq models (Bahdanau et al., 2015) would perform. Moreover, the performance of the word-based SMT model (M1) is surprising: for 7/10 languages it is the best submission by Parida et al. (2021), and it performs competitively with the best overall system for cni (25.8 vs. 25.3) This further indicates that the exploration of non-transformer models might yield interesting results. As indicated by the fact that ensembles (M3) are only used for one language (hch) by Knowles et al. (2021), ensembling does not seem to consistently result in better performance—but the question of how this depends on the potentially participating individual models remains open.

**Training Techniques:** Four different types of pretraining strategies prove effective: denoising (T5 style; TT1), denoising (mBART style; TT2), pretraining on backtranslated data (TT3), and pretraining a multilingual translation model (TT4)—either on a set of languages that includes the target language pair or on one that does not. However, they differ in their effectiveness: backtranslation seems to be particularly beneficial, given that it is part of the best system's training for 9/10 languages. While denoising helps as well, according to the references of the systems that use it, the effect is consistent, but rather small. Most systems employ finetuning on the target language pair as the last (or only) training step, to focus the model on the two languages of interest. Surprisingly, however, the winning system does not: it is a multilingual system with no specific emphasis on a specific language pair. This shows that finetuning is not indispensable. However, this leaves open the question of whether a final finetuning phase could have improved performance even further—or if it would instead hurt performance due to overfitting.

### 3.2. Natural language inference results

Results for this experiment are shown in Table 6. As expected, the zero-shot performance of XLM-R is extremely low for all 10 languages. The average accuracy when using English as the finetuning language is 38.48%, and 37.99% when using Spanish. Although one would expect finetuning on Spanish to achieve better performance, it only outperforms the English setting for 3 languages. Interestingly, performance is still above the random baseline for many languages, which may be due to English or Spanish entities found in the evaluation data. The best zero-shot performance is achieved for Nahuatl, with 42.59% accuracy using English, and for Quechua, with an accuracy of 39.51% when using Spanish. The lowest performance is 36.13% for Aymara and 35.73% for Rarámuri, when using English and Spanish, respectively.

**Table 6:** Accuracy of zero-shot, translate-train, and translate-test models.

	**aym**	**bzd**	**cni**	**gn**	**hch**	**nah**	**oto**	**quy**	**shp**	**tar**	**Avg**.
**Zero-shot**											
XLM-R (en)	36.13±0.88	39.65 ± 0.89	37.91 ± 0.82	39.47 ± 1.14	37.20 ± 1.32	42.59 ± 0.34	37.79 ± 0.78	37.24 ± 1.78	40.45 ± 0.89	36.36 ± 1.07	38.48 ± 1.05
XLM-R (es)	37.25 ± 2.33	39.38 ± 1.96	37.29 ± 1.12	39.25 ± 1.55	35.82 ± 1.01	38.98 ± 1.38	38.32 ± 1.47	39.51 ± 1.92	38.40 ± 0.87	35.73 ± 0.69	37.99 ± 1.51
**Zero-shot w/adaptation**											
XLM-R+MLM (en)	43.51 ± 1.69	38.13 ± 1.75	39.47 ± 1.19	52.44 ± 0.93	37.25 ± 2.60	46.21 ± 0.72	37.03 ± 3.28	61.78 ± 2.42	41.34 ± 0.61	39.82 ± 0.95	43.70 ± 1.83
XLM-R+MLM (es)	43.87 ± 0.14	40.05 ± 2.20	38.76 ± 0.08	52.27 ± 1.20	37.82 ± 1.59	44.17 ± 1.76	**40.55 ±** 1.07	**62.40 ±** 1.44	40.18 ± 0.95	38.45 ± 0.86	43.85 ± 1.30
**Translate-train**											
XLM-R (baseline)	**50.00 ±** 1.51	**51.42 ±** 1.24	**42.45 ±** 1.63	**58.89 ±** 2.70	**43.20 ±** 2.07	**55.33 ±** 1.12	36.01 ± 0.74	59.91 ± 0.20	**52.00 ±** 0.27	**42.04 ±** 1.81	**49.12 ±** 1.52
**Translate-test**											
XLM-R	39.73 ± 0.27	40.40 ± 0.13	34.71 ± 0.73	46.62 ± 2.29	38.00 ± 0.48	41.37 ± 0.16	35.29 ± 1.15	51.38 ± 1.24	39.51 ± 0.47	35.16 ± 0.97	40.22 ± 1.01

We train 3 models with different seeds and take the average as the final result for each language. The majority baseline represents the simple baseline of selecting the most frequent class. Random guessing would result in an accuracy of 33.33%. Standard deviations in the *Avg*. column represent pooled standard deviations. The bold values indicate the best results for each language.

Model adaptation using unlabeled data is helpful for this task. Finetuning on Spanish, along with adaptation, leads to an average performance gain of 5.86%, while finetuning on English leads to a gain of 5.22%. We see the largest performance gain for Quechua, with 24.53% better accuracy after continued pretraining. This is likely related to the amount of adaptation data: Quechua has the largest amount available, far surpassing the other languages.

Considering the translation-based approaches, we find that translating the training set greatly outperforms all other methods. Again, we see the largest performance gain between translate-train and the best non-adapted zero-shot model for Quechua, with a gain of 20.4%. At the other extreme, we see a performance decrease for Otomí, as compared to the zero-shot baseline. This performance gain is striking— translation quality as measured by ChrF and BLEU scores is quite low: the highest ChrF score is 0.33 and the highest BLEU score is 3.26. Although all the scores are low, we see a correlation between translation metrics and NLI performance: the Pearson correlation coefficient between ChrF score and translate-train performance is 0.82. On average, translate-train outperforms adapted models finetuned on English by 5.27%, and non-adapted models 10.64%.

The performance of translate-test is less impressive, only improving 1.74% over the best zero-shot baseline. It does not outperform model adaptation, which achieves a minimum of 3.48% improvement on average. We hypothesize that the reason translate-test does so poorly is that this method is highly sensitive to poor translation—if information in a test example is not captured during the translation, it becomes impossible for the model to make a prediction.

### 3.3. Analysis of translation-based approaches

**Figure 1:**
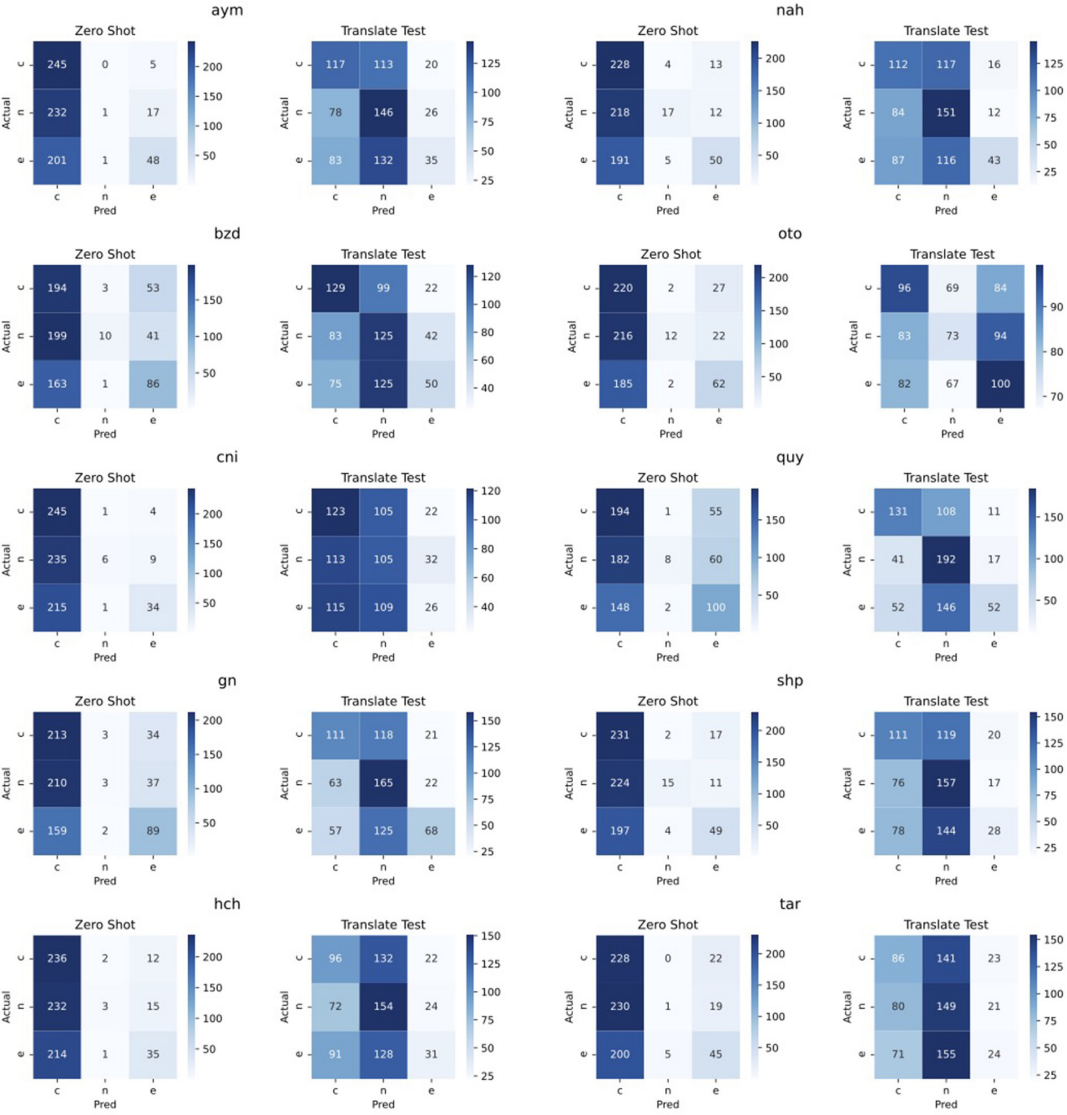
NLI confusion matrices for each language, comparing performance of the zero-shot baseline with translate-test. e, entailment; n, neutral; c, contradiction.

For this analysis, we focus on translate-test and present confusion matrices comparing this approach to the baseline in Figure 1. We can see that the baseline approach effectively always predicts an example as contradiction, except for some languages where it correctly detects a portion of the entailment examples (e.g., Bribri, Guarani, and Quechua). Effectively, the baseline approach never correctly predicts a neutral example. Now comparing the translate-test approach to the baseline, we can see a much more even distribution of predictions between neutral and contradiction, for all languages but Otomí, where predictions are mainly split between contradiction and entailment. Considering entailment examples, translate-train correctly classifies fewer examples with this label as compared to the baseline. These results are from the same trained model, and only the evaluation set is changed—since the translate-test model does not predict many examples as entailment, it may be possible that Spanish indicators of entailment are specifically being lost in translation, while indicators for examples such as contradiction are not. A further analysis to detect these indicators in the target languages may be helpful in identifying reasons for the poor performance of this method.

A qualitative analysis of the translate-test data shows that a common problem experienced for all languages is the extreme repetition of certain subwords. In an attempt to quantify this, we calculate the average number of characters and tokens per example, as a heuristic for poor quality. We calculate this for both the test set and its translations and present values in Table 7. We can see that for some languages, namely Bribri, Asháninka, and Otomí, the average number of characters in the premise is considerably larger than the standard test set. Interestingly, the number of tokens does not cleanly correlate with characters. Considering the hypothesis, we do not see the same drastic change after translation. This may indicate that the poor performance of translate-train is caused in part by failing to correctly translate the premise.

**Table 7:** Average length of an example, in both characters and tokens, for the collected test set as well as the translated test set used for translate-test.

**Lang**.	**Split**	**Premise**		**Hypothesis**	

		**Chars**	**Tokens**	**Chars**	**Tokens**
aym	Test	86.09	33.30	48.87	19.08
	Translate-test	89.74	23.58	60.74	15.37
bzd	Test	83.29	49.24	45.39	26.99
	Translate-test	145.83	39.06	57.99	15.38
cni	Test	87.06	29.59	51.45	17.28
	Translate-test	115.91	31.64	57.08	15.58
gn	Test	72.55	32.18	39.07	17.40
	Translate-test	71.57	18.50	42.27	10.69
hch	Test	91.31	38.55	52.54	22.09
	Translate-test	91.10	25.22	66.44	17.14
nah	Test	78.24	28.67	44.98	16.13
	Translate-test	77.79	20.76	43.58	11.65
oto	Test	85.94	36.99	45.62	19.65
	Translate-test	237.70	71.73	88.40	26.48
quy	Test	84.67	30.89	48.64	17.58
	Translate-test	74.11	19.30	46.32	11.72
shp	Test	92.47	30.72	49.56	16.11
	Translate-test	81.31	20.85	42.15	10.71
tar	Test	81.12	33.26	48.43	19.82
	Translate-test	79.68	22.48	64.93	17.74

## 4. Discussion

### 4.1. Pretrained models vs. translation-based approaches for semantic tasks

In this work, we present AmericasNLI, a multiway parallel NLI dataset covering 10 Indigenous languages of the Americas, which can be used to benchmark MT and NLI systems. Our aim is to answer the following RQs: (1) Do zero-shot approaches for NLI based on model adaptation perform well for truly low-resource languages, which are unseen to the underlying model? (2) How do translation-based approaches work for NLI if all we have are poor MT systems, and how do we build initial MT systems for the AmericasNLI languages? (3) Finally, how do translation-based and zero-shot approaches compare?

Conducting experiments with XLM-R, we find that the model's zero-shot performance is poor: it only slightly outperforms a majority baseline. This is in contrast to zero-shot results on the lower-level tasks of POS tagging and NER (Ebrahimi and Kann, 2021). While model performance can be improved by model adaptation *via* continued pretraining, we find that a translation-based approach—namely translate-train—outperforms all zero-shot approaches. Surprisingly, this is true even though the translation quality is quite low. It highlights that translation-based approaches might be promising ways to create human language technology for truly low-resource languages.

### 4.2. Shortcomings of natural language inference

From a usability standpoint, the task of NLI is useful in many ways. In terms of model implementation, the inputs are restricted to two sentences, and as a three-way classification task, model outputs are relatively easy to compute and handle (as compared to, e.g., a token-level classification or translation task). This relative simplicity allows for quick development of models and easier transfer between languages. The format of the inputs, as two separate, relatively short sentences, allows for quicker and more straightforward translation. Furthermore, it is easier for the meaning of each sentence to be preserved across translation when we are only considering sentences—in the case of, e.g., multiple choice question answering, a large paragraph containing background information, the question, and possible answers would need to be translated.

These benefits of NLI allow for a relatively rapid development of new, parallel evaluation sets through translation. However, they are also the main sources of drawbacks for the task. Considering the task specifically, having an input of two sentences can restrict how challenging the dataset can become. Additionally, with only three labels, it may not be clear, even to a human, how to classify the relation between the two sentences. Further, while MT makes translation quick and easy, translating also means that sentences in target languages are likely not to be representative of natural utterances spoken in those languages. As the original sources of the dataset were often conversational in nature, they may be fragments, may not always be grammatical, or may cover topics which are not commonly spoken about in the target language. Finally, regarding XNLI, which was created using crowdsourcing, prior work has found that artifacts created from the annotation process can allow models to cheat, inflating performance (Gururangan et al., 2018).

With these drawbacks in mind, however, the task of NLI still has value for measuring a model's ability to process a certain language, particularly when these languages do not have other evaluation datasets available. NLI can be used as a first step toward creating a suite of datasets for a language, although it should not be the end-all measure of a model's capabilities.

### 4.3. What is next for NLP for Indigenous languages?

Researchers who identify as Native American or LatinX are underrepresented in the machine learning and NLP communities. Relatedly, many truly low-resource languages, including Indigenous languages of the Americas, such as those in AmericasNLI, still receive worryingly little attention from researchers. This is unfortunate, since, according to Glottolog^12^, 86 language families and 95 language isolates can be found in the Americas, and many of them are labeled as endangered. The development of ML and NLP technologies has the potential to help keep them alive or support their revitalization (Mager et al., 2018b). In order to achieve real impact for Indigenous languages—including progress on problems that are of actual importance to the communities (https://doi.org/10.48550/arxiv.2206.00437), there is a need to unite more stakeholders, including native speakers, NLP researchers, linguists, ethicists, and industry professionals. NLP researchers specifically should investigate user needs and ensure that their research happens in close collaboration with the communities they expect to eventually use products that are based on their research findings.

From a technical perspective, considering the rather weak MT results in Section 3.1, it is obvious that MT systems for the AmericasNLI languages still have room for improvement. Possible ways to achieve such an improvement include data collection and data cleaning, the development of new and more suitable models, or the invention of better training and transfer learning algorithms. Besides the general benefits of strong MT systems for communities, the strong performance of translate-train in this work highlights that MT systems might also be the key to building systems for other NLP tasks of interest.

Given the typological differences between many Indigenous and high-resource languages, another RQ worth exploring is how to integrate more linguistic knowledge into machine learning systems for NLP. For instance, many Indigenous languages are morphologically rich (Kann et al., 2018; Mager et al., 2020). Explicit handling of morphology might result in better downstream-task systems.

### 4.4. Limitations of and ethical considerations regarding this work

In this work, we present AmericasNLI, an NLI dataset for 10 Indigenous languages of the Americas, which we create by translating an existing NLI dataset, XNLI Conneau et al. (2018). While this allows for results that are directly comparable to prior work on the original data as well as for the use of AmericasNLI for the evaluation of MT systems, it also means that this dataset inherits any biases and flaws which are contained in the previous dataset. Furthermore, research involving languages spoken by Indigenous communities raises ethical concerns regarding the exploitation of these languages and communities: It is crucial that the process does not exploit any member of the community or commodify the language (Schwartz, 2022). In addition, members of the community should directly benefit from the research. Translation for AmericasNLI was done by either paper authors or translators who were compensated at a rate based on the average rate for translation and the minimum wage in their country of residence. Additionally, many authors are members of and/or have a record of close work with communities who speak a language contained in AmericasNLI.

## Data availability statement

The datasets presented in this study can be found in online repositories. The names of the repository/repositories and accession number(s) can be found below: https://nala-cub.github.io/resources/.

## Author contributions

KK, AE, MM, and AO led the effort. AE conducted the experiments. JO, AE, AF, VC, LC, GG-L, RR, EM, and RC-S were involved in the data collection. KK and AE wrote the majority of the paper. All authors provided input and feedback on the manuscript.

## Funding

This research was made possible by financial support from Facebook AI Research, Microsoft Research, Google Research, the Institute of Computational Linguistics at the University of Zurich, the NAACL Emerging Regions Fund, Comunidad Elotl, and Snorkel AI.

## Conflict of interest

AF was employed by Facebook AI Research. VC was employed by Microsoft Turing Research. The remaining authors declare that the research was conducted in the absence of any commercial or financial relationships that could be construed as a potential conflict of interest.

## Publisher's note

All claims expressed in this article are solely those of the authors and do not necessarily represent those of their affiliated organizations, or those of the publisher, the editors and the reviewers. Any product that may be evaluated in this article, or claim that may be made by its manufacturer, is not guaranteed or endorsed by the publisher.

## Footnotes

1 https://bdpi.cultura.gob.pe/pueblos/ashaninka

2 https://www.inec.cr/social/grupos-etnicos-raciales

3 https://www.ine.gov.py/news/25-de-agosto-dia-del-Idioma-Guarani.php

4 https://es.wikipedia.org/wiki/Idioma_guarani

5 https://site.inali.gob.mx/Micrositios/normas/pdf/Norma_Otomi.pdf

6 https://www.gob.mx/inpi/articulos/etnografia-del-pueblo-tarahumara-raramuri

7 https://www.inali.gob.mx/pdf/CLIN_completo.pdf

8 https://www.gob.mx/cms/uploads/attachment/file/59310/catalogo_lenguas_indigenas_mexico_2008.pdf

9 While Spanish is the featured high-resource language in our experiments, AmericasNLI is designed in such a way that is can be used to evaluate MT between the 10 Indigenous languages and any of the 15 high-/medium-resource languages in XNLI.

10 Otomí online corpus: https://tsunkua.elotl.mx/about/.

11 We note that language similarity is not a well-defined concept, and many factors may contribute to a model's performance for a given language.

12 https://glottolog.org

## References

[B1] Agić Ž. Johannsen A. Plank B. Martínez Alonso H. Schluter N. Søgaard A. (2016). Multilingual projection for parsing truly low-resource languages. Trans. Assoc. Comput. Linguist. 4, 100. 10.1162/tacl_a_00100

[B2] Agić Ž. Vulić I. (2019). JW300: a wide-coverage parallel corpus for low-resource languages, in Proceedings of the 57th Annual Meeting of the Association for Computational Linguistics (Florence: Association for Computational Linguistics).

[B3] Alabbas M. (2013). A dataset for Arabic textual entailment, in Proceedings of the Student Research Workshop Associated With RANLP 2013 (Hissar: INCOMA Ltd. Shoumen, BULGARIA), 7–13.

[B4] Amirkhani H. AzariJafari M. Amirak A. Pourjafari Z. Jahromi S. F. Kouhkan Z. (2020). Farstail: a persian natural language inference dataset. arXiv[Preprint].arXiv:2009.08820. 10.48550/arXiv.2009.08820

[B5] Ammar W. Mulcaire G. Tsvetkov Y. Lample G. Dyer C. Smith N. A. (2016). Massively multilingual word embeddings. ArXiv, abs/1602.01925. 10.48550/arXiv.1602.01925

[B6] Artetxe M. Schwenk H. (2019). Massively multilingual sentence embeddings for zero-shot cross-lingual transfer and beyond. Trans. Assoc. Comput. Linguist. 7, 597–610. 10.1162/tacl_a_00288

[B7] Bahdanau D. Cho K. Bengio Y. (2015). Neural machine translation by jointly learning to align and translate, in Proceedings of the International Conference on Learning Representations (San Diego, CA).

[B8] Bojanowski P. Grave E. Joulin A. Mikolov T. (2017). Enriching word vectors with subword information. Trans. Assoc. Comput. Linguist. 5, 51. 10.1162/tacl_a_00051

[B9] Bojar O. Buck C. Federmann C. Haddow B. Koehn P. Leveling J.. (2014). Findings of the 2014 workshop on statistical machine translation, in Proceedings of the Ninth Workshop on Statistical Machine Translation (Baltimore, MD: Association for Computational Linguistics).

[B10] Bollmann M. Aralikatte R. Murrieta Bello H. Hershcovich D. de Lhoneux M. Søgaard A. (2021). Moses and the character-based random babbling baseline: CoAStaL at AmericasNLP 2021 shared task, in Proceedings of the First Workshop on Natural Language Processing for Indigenous Languages of the Americas (Association for Computational Linguistics), 248–254. 10.18653/v1/2021.americasnlp-1.28

[B11] Bos J. Zanzotto F. M. Pennacchiotti M. (2009). Textual entailment at EVALITA 2009, in Proceedings of EVALITA 2009 (Reggio Emilia).

[B12] Bowman S. R. Angeli G. Potts C. Manning C. D. (2015). A large annotated corpus for learning natural language inference, in Proceedings of the 2015 Conference on Empirical Methods in Natural Language Processing (EMNLP) (Lisbon: Association for Computational Linguistics).

[B13] Brambila D. (1976). Diccionario rarámuri-castellano Tarahumar. Obra Nacional de la buena Prensa.

[B14] Brown P. F. Della Pietra S. A. Della Pietra V. J. Mercer R. L. (1993). The mathematics of statistical machine translation: parameter estimation. Computat. Linguist. 19, 263–311.

[B15] Caballero G. (2008). Choguita Raramuri Tarahumara phonology and morphology (Ph. D. thesis). University of California, Berkeley, CA, United States.

[B16] Cajero M. (1998). Raíces del Otomí: diccionario. Gobierno del Estado de Tlaxcala.

[B17] Cajero M. (2009). Historia de los Otomíes en Ixtenco, Vol. 1. Tlaxcala: Instituto Tlaxcalteca de la Cultura.

[B18] Campbell L. (2000). American INDIAN Languages: The Historical Linguistics of Native America. Oxford: Oxford University Press.

[B19] Chau E. C. Lin L. H. Smith N. A. (2020). Parsing with multilingual BERT, a small corpus, and a small treebank, in Findings of the Association for Computational Linguistics: EMNLP 2020 (Association for Computational Linguistics), 1324–1334. 10.18653/v1/2020.findings-emnlp.118

[B20] Chiruzzo L. Amarilla P. Ríos A. Giménez Lugo G. (2020). Development of a Guarani-Spanish parallel corpus, in Proceedings of the 12th Language Resources and Evaluation Conference (Marseille: European Language Resources Association), 2629–2633.

[B21] Conneau A. Khandelwal K. Goyal N. Chaudhary V. Wenzek G. Guzmán F.. (2020). Unsupervised cross-lingual representation learning at scale, in Proceedings of the 58th Annual Meeting of the Association for Computational Linguistics (Association for Computational Linguistics), 8440–8451. 10.18653/v1/2020.acl-main.747

[B22] Conneau A. Lample G. (2019). Cross-lingual language model pretraining, in Proceedings of the Conference on Neural Information Processing Systems (Vancouver, BC).

[B23] Conneau A. Rinott R. Lample G. Williams A. Bowman S. R. Schwenk H.. (2018). Xnli: evaluating cross-lingual sentence representations, in Proceedings of the 2018 Conference on Empirical Methods in Natural Language Processing (Brussels: Association for Computational Linguistics).

[B24] Constenla A. Elizondo F. Pereira F. (2004). Curso Básico de Bribri. Editorial de la Universidad de Costa Rica.

[B25] Devlin J. Chang M.-W. Lee K. Toutanova K. (2019). BERT: pre-training of deep bidirectional transformers for language understanding, in Proceedings of the 2019 Conference of the North American Chapter of the Association for Computational Linguistics: Human Language Technologies, Volume 1 (Long and Short Papers) (Minneapolis, MN. Association for Computational Linguistics).

[B26] Dos Santos R. A. (2017). Diglossia No Paraguai: A restrição dos monolíngues em guarani no acesso à informação, in Trabalho de Conclusão de Curso, Bacharelado em Línguas Estrangeiras (Brasilia: Universidade de Brasília).

[B27] Ebrahimi A. Kann K. (2021). How to adapt your pretrained multilingual model to 1600 languages, in Proceedings of the 59th Annual Meeting of the Association for Computational Linguistics and the 11th International Joint Conference on Natural Language Processing (Volume 1: Long Papers) (Association for Computational Linguistics), 4555–4567. 10.18653/v1/2021.acl-long.351

[B28] Eichler K. Gabryszak A. Neumann G. (2014). An analysis of textual inference in German customer emails, in Proceedings of the Third Joint Conference on Lexical and Computational Semantics (*SEM 2014) (Dublin: Association for Computational Linguistics; Dublin City University), 69–74. 10.3115/v1/S14-1009

[B29] Enrique M. P. (2005). Diccionario Fraseológico Bribri-Espanol~ Espanol-Bribri, 2nd Edn~. San Jose: Editorial de la Universidad de Costa Rica.

[B30] Eskander R. Muresan S. Collins M. (2020). Unsupervised cross-lingual part-of-speech tagging for truly low-resource scenarios, in Proceedings of the 2020 Conference on Empirical Methods in Natural Language Processing (EMNLP) (Association for Computational Linguistics), 4820–4831. 10.18653/v1/2020.emnlp-main.391

[B31] Fang Y. Wang S. Gan Z. Sun S. Liu J. (2021). Filter: an enhanced fusion method for cross-lingual language understanding. Proc. AAAI Conf. Artif. Intell. 35, 12776–12784. 10.1609/aaai.v35i14.17512

[B32] Faust N. (1973). Lecciones para el Aprendizaje del idioma shipibo-conibo, Volume 1 of Documento de Trabajo. Yarinacocha: Instituto Lingüístico de Verano.

[B33] Feldman I. Coto-Solano R. (2020). Neural machine translation models with back-translation for the extremely low-resource indigenous language Bribri, in Proceedings of the 28th International Conference on Computational Linguistics (Barcelona: International Committee on Computational Linguistics).

[B34] Flores Solórzano S. (2017). Corpus Oral Pandialectal de la Lengua Bribri. Available online at: bribri.net

[B35] Galarreta A.-P. Melgar A. Oncevay A. (2017). Corpus creation and initial SMT experiments between Spanish and Shipibo-konibo, in Proceedings of the International Conference Recent Advances in Natural Language Processing, RANLP 2017 (Varna: INCOMA Ltd.).

[B36] Goddard I. (1996). Introduccion, in Handbook of North American Indians, Vol. 17, Chapter 1, ed Sturtevant W. C. (Austin, TX: University of Texas), 1–6.

[B37] Gómez Montoya H. E. Rojas K. D. R. Oncevay A. (2019). A continuous improvement framework of machine translation for Shipibo-konibo, in Proceedings of the 2nd Workshop on Technologies for MT of Low Resource Languages (Dublin: European Association for Machine Translation), 17–23.

[B38] Grave E. Bojanowski P. Gupta P. Joulin A. Mikolov T. (2018). Learning word vectors for 157 languages, in Proceedings of the International Conference on Language Resources and Evaluation (Miyazaki).

[B39] Greenberg J. H. (1963). Universals of language MIT press.

[B40] Gururangan S. Swayamdipta S. Levy O. Schwartz R. Bowman S. Smith N. A. (2018). Annotation artifacts in natural language inference data, in Proceedings of the 2018 Conference of the North American Chapter of the Association for Computational Linguistics: Human Language Technologies, Volume 2 (Short Papers) (New Orleans, LA: Association for Computational Linguistics).

[B41] Gutierrez-Vasques X. Sierra G. Pompa I. H. (2016). Axolotl: a web accessible parallel corpus for Spanish-Nahuatl, in Proceedings of the Tenth International Conference on Language Resources and Evaluation (LREC'16) (Slovenia: European Language Resources Association, ELRA), 4210–4214.

[B42] Homola P. (2012). Building a formal grammar for a polysynthetic language, in Formal Grammar, eds de Groote P. Nederhof M. -J. (Berlin; Heidelberg: Springer Berlin Heidelberg), 228–242.

[B43] Huarcaya Taquiri D. (2020). Traducción automática neuronal para lengua nativa peruana (Bachelor's thesis). Universidad Peruana Unión.

[B44] INEC (2011). Población total en territorios indígenas por autoidentificación a la etnia indígena y habla de alguna lengua indígena, según pueblo y territorio indígena, in Censo 2011, ed de Estadística y Censos I. N.

[B45] Iturrioz J. L. Gómez-López P. (2008). Gramatica wixarika. Lincom Studies in Native American Linguistics.

[B46] Jara Murillo C. V. García Segura A. (2013). Se' ttö' bribri ie Hablemos en Bribri. EDigital.

[B47] Johnson M. Schuster M. Le Q. Krikun M. Wu Y. Chen Z.. (2017). Google's multilingual neural machine translation system: enabling zero-shot translation. Trans. Assoc. Comput. Linguist. 5, 339–351. 10.1162/tacl_a_00065

[B48] Kann K. Mager Hois J. M. Meza-Ruiz I. V. Schütze H. (2018). Fortification of neural morphological segmentation models for polysynthetic minimal-resource languages, in Proceedings of the 2018 Conference of the North American Chapter of the Association for Computational Linguistics: Human Language Technologies, Volume 1 (Long Papers) (New Orleans, LA: Association for Computational Linguistics).

[B49] Knowles R. Stewart D. Larkin S. Littell P. (2021). NRC-CNRC machine translation systems for the 2021 AmericasNLP shared task, in Proceedings of the First Workshop on Natural Language Processing for Indigenous Languages of the Americas (Association for Computational Linguistics), 224–233. 10.18653/v1/2021.americasnlp-1.25

[B50] Kudo T. Richardson J. (2018). SentencePiece: a simple and language independent subword tokenizer and detokenizer for neural text processing, in Proceedings of the 2018 Conference on Empirical Methods in Natural Language Processing: System Demonstrations (Brussels: Association for Computational Linguistics).

[B51] Lampie G. Conneau A. Denoyer L. Ranzato M. (2018a). Unsupervised machine translation using monolingual corpora only, in International Conference on Learning Representations (Vancouver, BC).

[B52] Lample G. Conneau A. Ranzato M. Denoyer L. Jégou H. (2018b). Word translation without parallel data, in 6th International Conference on Learning Representations, ICLR 2018, Vancouver, BC, Canada, April 30 - May 3, 2018 (Vancouver, BC: Conference Track Proceedings).

[B53] Lauscher A. Ravishankar V. Vulić I. Glavaš G. (2020). From zero to hero: on the limitations of zero-shot language transfer with multilingual transformers, in Proceedings of the 2020 Conference on Empirical Methods in Natural Language Processing (EMNLP) (Online. Association for Computational Linguistics).

[B54] Liu Y. Gu J. Goyal N. Li X. Edunov S. Ghazvininejad M.. (2020). Multilingual denoising pre-training for neural machine translation. Trans. Assoc. Comput. Linguist. 8, 726–742. 10.1162/tacl_a_00343

[B55] Loriot J. Lauriault E. Day D. de Educación P. M. (1993). Diccionario Shipibo-Castellano. Instituto Linguistico de Verano.

[B56] Lumholtz C. (2011). Unknown Mexico: A Record of Five Years' Exploration Among the Tribes of the Western Sierra Madre, volume 2. Cambridge: Cambridge University Press.

[B57] MacSwan J. (1998). The argument status of nps in southeast puebla nahuatl: Comments on the polysynthesis parameter. Southwest J. Linguist. 17, 101–114.

[B58] Mager M. Carrillo D. Meza I. (2018a). Probabilistic finite-state morphological segmenter for wixarika (huichol) language. J. Intell. Fuzzy Syst. 34, 3081–3087. 10.3233/JIFS-169492

[B59] Mager M. Çetinoğlu Ö. Kann K. (2020). Tackling the low-resource challenge for canonical segmentation. arXiv preprint arXiv:2010.02804. 10.18653/v1/2020.emnlp-main.423

[B60] Mager M. Gutierrez-Vasques X. Sierra G. Meza-Ruiz I. (2018b). Challenges of language technologies for the indigenous languages of the Americas, in Proceedings of the 27th International Conference on Computational Linguistics (Santa Fe, NM: Association for Computational Linguistics), 55–69.

[B61] Mager M. Oncevay A. Ebrahimi A. Ortega J. Rios A. Fan A.. (2021). Findings of the AmericasNLP 2021 shared task on open machine translation for indigenous languages of the Americas, in Proceedings of the First Workshop on Natural Language Processing for Indigenous Languages of the Americas (Association for Computational Linguistics).

[B62] Mager-Hois J. M. (2017). Traductor hıbrido wixárika-espanol con escasos recursos bilingües (Ph.D. thesis, Master's thesis). Universidad Autónoma Metropolitana.

[B63] McCarthy A. D. Wicks R. Lewis D. Mueller A. Wu W. Adams O.. (2020). The Johns Hopkins University Bible corpus: 1600+ tongues for typological exploration, in Proceedings of the 12th Language Resources and Evaluation Conference (Marseille: European Language Resources Association), 2884–2892.

[B64] Melià B. (1992). La lengua Guaraní del Paraguay: Historia, sociedad y literatura. Madrid: Editorial MAPFRE.

[B65] Mihas E. (2011). Añaani Katonkosatzi Parenini, El idioma del alto Perené. Milwaukee, WI: Clarks Graphics.

[B66] Mihas E. (2017). The kampa subgroup of the arawak language family, in The Cambridge Handbook of Linguistic Typology, eds Aikhenvald A. Dixon R. Cambridge Handbooks in Language and Linguistics (Cambridge: Cambridge University Press), 782–814.

[B67] Mikolov T. Sutskever I. Chen K. Corrado G. S. Dean J. (2013). Distributed representations of words and phrases and their compositionality, in Advances in Neural Information Processing Systems, Vol. 26, eds Burges C. J. C. Bottou L. Welling M. Ghahramani Z. Weinberger K. Q. (Red Hook, NY: Curran Associates, Inc.), 3111–3119.

[B68] Moreno O. (2021). The repucs spanish quechua submission to the AmericasNLP 2021 shared task on open machine translation, in Proceedings of the First Workshop on Natural Language Processing for Indigenous Languages of the Americas (Association for Computational Linguistics), 241–247. 10.18653/v1/2021.americasnlp-1.27

[B69] Moseley C. (2010). Atlas of the World's Languages in Danger. Unesco.

[B70] Muller B. Anastasopoulos A. Sagot B. Seddah D. (2020). When being unseen from mbert is just the beginning: Handling new languages with multilingual language models. ArXiv, abs/2010.12858. 10.18653/v1/2021.naacl-main.38

[B71] Murillo J. Victoria C (2018b). *I Tte* Historias Bribris, 2nd Edǹ. Editorial de la Universidad de Costa Rica.

[B72] Murillo J. Victoria C. (2018a). Gramática de la Lengua Bribri. EDigital.

[B73] Nagoudi E. M. B. Chen W.-R. Abdul-Mageed M. Cavusoglu H. (2021). IndT5: a text-to-text transformer for 10 indigenous languages, in Proceedings of the First Workshop on Natural Language Processing for Indigenous Languages of the Americas (Association for Computational Linguistics), 265–271. 10.18653/v1/2021.americasnlp-1.30

[B74] Nichols J. (1986). Head-marking and dependent-marking grammar. Language 62, 56–119. 10.1353/lan.1986.0014

[B75] Ortega J. Castro-Mamani R. A. Montoya Samame J. R. (2020). Overcoming resistance: the normalization of an Amazonian tribal language, in Proceedings of the 3rd Workshop on Technologies for MT of Low Resource Languages (Suzhou: Association for Computational Linguistics), 1–13.

[B76] Parida S. Panda S. Dash A. Villatoro-Tello E. Doğruöz A. S. Ortega-Mendoza R. M.. (2021). Open machine translation for low resource South American languages (AmericasNLP 2021 shared task contribution), in Proceedings of the First Workshop on Natural Language Processing for Indigenous Languages of the Americas (Association for Computational Linguistics), 218–223. 10.18653/v1/2021.americasnlp-1.24

[B77] Pennington J. Socher R. Manning C. (2014). GloVe: global vectors for word representation, in Proceedings of the 2014 Conference on Empirical Methods in Natural Language Processing (EMNLP) (Doha: Association for Computational Linguistics).

[B78] Peters M. E. Neumann M. Iyyer M. Gardner M. Clark C. Lee K.. (2018). Deep contextualized word representations, in Proceedings of the 2018 Conference of the North American Chapter of the Association for Computational Linguistics: Human Language Technologies, Volume 1 (Long Papers) (New Orleans, LA: Association for Computational Linguistics).

[B79] Pfeiffer J. Vuli,ć I. Gurevych I. Ruder S. (2020a). Unks everywhere: Adapting multilingual language models to new scripts, in Proceedings of the 2021 Conference on Empirical Methods in Natural Language Processing (Punta Cana: Association for Computational Linguistics).

[B80] Pfeiffer J. Vulic I. Gurevych I. Ruder S. (2020b). MAD-X: An adapter-based framework for ulti-task cross-lingual transfer, in Proceedings of the 2020 Conference on Empirical Methods in Natural Language Processing (Association for Computational Linguistics), 7654–7673. 10.18653/v1/2020.emnlp-main.617

[B81] Prokopidis P. Papavassiliou V. Piperidis S. (2016). Parallel global voices: a collection of multilingual corpora with citizen media stories, in Proceedings of the Tenth International Conference on Language Resources and Evaluation (LREC'16) (Portorov: European Language Resources Association, ELRA), 900–905.

[B82] Raffel C. Shazeer N. Roberts A. Lee K. Narang S. Matena M.. (2020). Exploring the limits of transfer learning with a unified text-to-text transformer. J. Mach. Learn. Res. 21, 1–67. 10.48550/arXiv.1910.1068334305477

[B83] Romano, Rubén and Richer, Sebastián. (2008). Ñaantsipeta Asháninkaki Birakochaki. Available online at: www.lengamer.org/publicaciones/diccionarios/

[B84] Ruder S. Constant N. Botha J. Siddhant A. Firat O. Fu J.. (2021). XTREME-R: towards more challenging and nuanced multilingual evaluation, in Proceedings of the 2021 Conference on Empirical Methods in Natural Language Processing (Punta Cana: Association for Computational Linguistics).

[B85] Sánchez Avendaño C. (2013). Lenguas en peligro en Costa Rica: vitalidad, documentación y descripción. Revista Káñina 37, 219–250. 10.15517/rk.v37i1.10589

[B86] Schwartz L. (2022). Primum Non Nocere: before working with Indigenous data, the ACL must confront ongoing colonialism, in Proceedings of the 60th Annual Meeting of the Association for Computational Linguistics (Volume 2: Short Papers) (Dublin: Association for Computational Linguistics).

[B87] SEGOB (2020a). Sistema de Información Cultural-Lenguas Indígenas: Huichol. Available online at: https://sic.gob.mx/ficha.php?table=inali_li

[B88] SEGOB (2020b). Sistema de Información Cultural-Lenguas indígenas: Nnahuatl. Available online at: https://sic.gob.mx/ficha.php?table=inali_liandamp;table_id=5

[B89] SEGOB (2020c). Sistema de Información Cultural-Lenguas Indígenas: Tarahumara. Available online at: http://sic.gob.mx/ficha.php?table=inali_liandamp;table_id=15

[B90] Sennrich R. Haddow B. Birch A. (2016). Improving neural machine translation models with monolingual data, in Proceedings of the 54th Annual Meeting of the Association for Computational Linguistics (Volume 1: Long Papers) (Berlin: Association for Computational Linguistics).

[B91] Tiedemann J. (2012). Parallel data, tools and interfaces in OPUS, in Proceedings of the Eighth International Conference on Language Resources and Evaluation (LREC'12) (Turkey: European Language Resources Association, ELRA), 2214–2218. Available online at: http://www.lrec-conf.org/proceedings/lrec2012/pdf/463_Paper.pdf

[B92] Valenzuela P. (2003). Transitivity in Shipibo-Konibo grammar (Ph.D. thesis). University of Oregon.

[B93] Vasquez A. Ego Aguirre R. Angulo C. Miller J. Villanueva C. Agić Z.. (2018). Toward universal dependencies for shipibo-konibo, in Proceedings of the Second Workshop on Universal Dependencies (UDW 2018) (Brussels: Association for Computational Linguistics).

[B94] Vaswani A. Shazeer N. Parmar N. Uszkoreit J. Jones L. Gomez A. N.. (2017). Attention is all you need, in Advances in Neural Information Processing Systems, Vol. 30, eds Guyon I. Luxburg U. V. Bengio S. Wallach H. Fergus R. Vishwanathan S. Garnett R. (Red Hook, NY: Curran Associates, Inc.).

[B95] Vázquez R. Scherrer Y. Virpioja S. Tiedemann J. (2021). The Helsinki submission to the AmericasNLP shared task, in Proceedings of the First Workshop on Natural Language Processing for Indigenous Languages of the Americas (Association for Computational Linguistics).

[B96] Wang Z. K K. Mayhew S. Roth D. (2020). Extending multilingual BERT to low-resource languages, in Findings of the Association for Computational Linguistics: EMNLP 2020 (Association for Computational Linguistics), 2649–2656. 10.18653/v1/2020.findings-emnlp.240

[B97] Williams A. Nangia N. Bowman S. (2018). A broad-coverage challenge corpus for sentence understanding through inference, in Proceedings of the 2018 Conference of the North American Chapter of the Association for Computational Linguistics: Human Language Technologies, Volume 1 (Long Papers) (New Orleans, LA: Association for Computational Linguistics), 1112–1122. 10.18653/v1/N18-1101

[B98] Yarowsky D. Ngai G. Wicentowski R. (2001). Inducing multilingual text analysis tools *via* robust projection across aligned corpora, in Proceedings of the First International Conference on Human Language Technology Research (San Diego, CA). Available online at: https://aclanthology.org/H01-1035

[B99] Zheng F. Reid M. Marrese-Taylor E. Matsuo Y. (2021). Low-resource machine translation using cross-lingual language model pretraining, in Proceedings of the First Workshop on Natural Language Processing for Indigenous Languages of the Americas (Association for Computational Linguistics), 234–240. 10.18653/v1/2021.americasnlp-1.26

